# Long term evolution of endothelial function during kidney transplantation

**DOI:** 10.1186/s12882-016-0369-5

**Published:** 2016-10-22

**Authors:** Clark Kensinger, Aihua Bian, Meagan Fairchild, Guanhua Chen, Loren Lipworth, T. Alp Ikizler, Kelly A. Birdwell

**Affiliations:** 1 Department of Surgery, Vanderbilt University Medical Center, 1161 21st Avenue South, D4313 MCN, Nashville, TN 37232 USA; 2 Department of Biostatistics, Vanderbilt University Medical Center, 2525 West End Avenue, Suite 11000, Nashville, TN 37203 USA; 3 Division of Nephrology and Hypertension, Vanderbilt University Medical Center, 1161 21st Avenue, S-3223 MCN, Nashville, TN 37232 USA; 4 Division of Epidemiology, Department of Medicine, Vanderbilt University Medical Center, 2525 West End, Suite 600, Nashville, TN 37203 USA; 5 Department of Medicine, Division of Nephrology and Hypertension, Vanderbilt University Medical Center, 1161 21st Avenue, S-3223 MCN, Nashville, TN 34232 USA

**Keywords:** Cardiovascular disease, Cardiovascular risk factors, Endothelial dysfunction, Flow-mediated dilation, Kidney transplantation

## Abstract

**Background:**

Endothelial dysfunction is an important precursor to the development of atherosclerosis, and has been suggested to play a role in the increased cardiovascular risk in patients with end stage renal disease. Endothelial function improves rapidly following post kidney transplantation, but the long term change remains unclear. Hypothesizing that endothelial function would remain improved long term post kidney transplantation, we evaluated the longitudinal change of endothelial function, measured by flow-mediated dilation (FMD) of the brachial artery, from months 1 to 24 post transplantation. Given the previously reported association of fibroblast growth factor 23 (FGF-23) with endothelial dysfunction, we also examined changes in the association between FGF-23 levels and the change in FMD following kidney transplantation.

**Methods:**

We performed a prospective cohort study of 149 kidney transplant recipients, measuring endothelial function by FMD at months 1, 12, and 24 post-transplant. FGF-23 levels were measured at months 1 and 24 post-transplant. Linear mixed effects models were used to assess both the unadjusted and adjusted outcomes.

**Results:**

The cohort (mean age 49 ± 13 years) was 74 % male and 75 % white. The median FMD was 6.3 % (IQR: 3.4, 10.2), 5.4 % (IQR: 3.1, 8.5), and 5.6 % (IQR: 3.5, 9.1) at 1, 12, and 24 months, respectively. After adjustment for covariates, compared to month 1, no change occurred in FMD at 12 months (−0.66 %; 95 % CI: −1.81 %, 0.49 %; *P* = 0.262) or 24 months (−0.25 %; 95%CI: −1.76 %, 1.26 %; *P* = 0.746). FGF-23 decreased significantly over time (*P* = 0.024), but there was no significant association between FGF-23 and FMD (*P* = 0.799).

**Conclusion:**

Endothelial function remained stable at 12 and 24 months from 1 month post-kidney transplant, indicating that the improved endothelial function seen with transplant is maintained up to 2 years post transplantation. There was also no significant association between FGF-23 and endothelial function following kidney transplantation.

## Background

Patients with end stage renal disease (ESRD) requiring maintenance dialysis experience a high incidence and prevalence of cardiovascular disease, which is consequently a leading cause of mortality in this patient population [1]. Successful kidney transplantation is associated with lower cardiovascular morbidity and mortality compared to patients who remain on the transplant wait list [2–4]. Despite improvement in traditional cardiovascular risk factors following kidney transplantation, the death rate from cardiovascular disease in patients with a functioning graft remains high compared to the general population [5]. Understanding the progression of unique cardiovascular risk factors associated with kidney transplantation may provide potential opportunities for risk modification leading to improved survival.

One marker of increased cardiovascular risk is impairment of endothelial function, which is an important precursor to the development of atherosclerosis. Impaired brachial artery endothelial function serves as a marker of underlying atherosclerotic risk and is a reliable surrogate for coronary endothelial function [6]. Several previous studies have reported impaired endothelial function in patients with chronic kidney disease [7–10]. Endothelial dysfunction is universally present in dialysis patients, and it can predict cardiovascular events in patients with kidney disease [7, 8, 11, 12]. Several studies have shown that endothelial function improves immediately post-transplant compared to pre-transplant [10, 13, 14]. However, no long term study of repeated endothelial function assessment has been performed in medical stable kidney transplant recipients.

A high concentration of circulating Fibroblast Growth Factor 23 (FGF-23) is also an independent risk factor for kidney disease progression, cardiovascular disease and mortality [15–21]. Fibroblast Growth Factor 23 is linked to endothelial dysfunction in patients with ESRD [19, 22–24] and FGF-23 levels have been shown to significantly decrease following successful kidney transplantation [15]. However, the degree to which endothelial function changes in conjunction with decreasing levels of FGF-23 following kidney transplant remains unclear.

Our aim was to evaluate endothelial dysfunction longitudinally, as measured by flow-mediated dilation (FMD), in a cohort of medically stable, kidney transplant recipients starting at 1 month post transplantation. In addition, we evaluated FGF-23 concentration following transplant to investigate a possible association between FGF-23 levels and changes in endothelial function. We hypothesized that long-term endothelial function would remain improved following successful kidney transplantation, and the longitudinal reduction in FGF-23 levels in the setting of a functioning allograft would be associated with improved endothelial function.

## Methods

### Study design

We conducted a single-center, prospective cohort study. De novo kidney transplant recipients were recruited from the Vanderbilt University Medical Center (VUMC) Renal Transplant Clinic from August 2009 through May 2013. Inclusion criteria included patients aged ≥ 18 years who were undergoing or had recently undergone kidney transplantation. There were no exclusion criteria. Consent for participation in the study was obtained prior to discharge from the hospital following kidney transplant. Given the widely dispersed geographic area of patients on the kidney transplant waiting list at VUMC, obtaining a baseline FMD measurement prior to kidney transplantation was not logistically practical, thus patients were first evaluated at 1 month following kidney transplantation. Additional follow up visits were performed at 12 and 24 months. All patients gave informed consent and the VUMC Institutional Review Board approved the study protocol.

### Flow-mediated dilation protocol

A single, licensed sonographer blinded to clinical details and previous FMD measurements of the study participants obtained the ultrasound images and performed the FMD study. The initial FMD was measured at 1 month following transplant. Follow up FMD examinations were performed at 12 and 24 months.

Flow-mediated dilation was performed as originally described by Celermajer and colleagues [25]. Endothelial function of the brachial artery was measured following ischemic reactive hyperemia to determine the resulting FMD. Each patient in the cohort was requested not to eat, drink (including alcohol, caffeine, etc.), or smoke for 12 h prior to the examination. The criteria to begin the study required a patient’s systolic blood pressure to be stable within 5 mmHg over several, separate recordings performed at two-minute intervals. A longitudinal image of the brachial artery was taken in the supine position at rest with a high-resolution ultrasound to obtain the baseline diameter *(10.0 MHz linear array transducer, Philips iU 22, Andover, MA).* A pneumatic tourniquet was inflated around the lower arm distal to the site at which the baseline brachial artery diameter was measured. The tourniquet was inflated to 250 mmHg for a total time of 5 min. Reactive hyperemia was measured after restoration of blood flow following the release of the pneumatic blood pressure cuff. Brachial artery diameter was measured at 60 s and 90 s following the release of the blood pressure cuff. Arterial diameter was measured from the media-adventitia interface.

The FMD studies were evaluated by one reviewer (CK) for consistency and accuracy of the brachial artery diameter measurements at the 60 and 90 s time points following the induction of hyperemia after the deflation of the pneumatic tourniquet.

### Flow-mediated dilation calculation

Endothelium-dependent FMD was expressed as the percentage change of the brachial artery diameter from baseline to the diameter following reactive hyperemia (Post-test diameter-Baseline diameter)/Baseline diameter). The larger percent change of the two time points measured (60 or 90 s) served as the reported FMD. An increase in FMD over time indicates improved endothelial function. An acceptable reproducibility during longitudinal evaluations is a mean difference of less than 2–3 % [26]. Therefore, we defined an improvement in FMD of more than 3 % as necessary to detect a treatment benefit following kidney transplantation [27]. We also defined normal endothelial function as a FMD greater than 6.3 % [28].

### Fibroblast growth factor-23 measurement

Serum concentrations of FGF-23 were measured in duplicate after a single thaw of stored blood specimens using an intact FGF-23 ELISA kit (Immutopics, San Clemente, CA). Measurements in blood samples obtained at 1 month following kidney transplant served as the initial FGF-23 level. The longitudinal assessment of FGF-23 levels was performed on blood samples obtained at the 24-month follow-up. The coefficient of determination from the primary standard deviation curve for the FGF-23 measurements was 0.9999, indicating high reproducibility.

### Outcomes

The primary outcome was defined as the change in FMD from the one-month post kidney transplant evaluation to the 12-month follow-up. Secondary outcomes include the change in FMD from 1 month to 24 months following kidney transplant, change in FMD from 12 months to 24 months following kidney transplant, and change in FGF-23 from 1 month to 24 months post-kidney transplant. We also examined the association between FGF-23 levels and the progression of FMD from 1 month to 24 months following kidney transplantation.

### Statistical analysis

Descriptive statistics were presented as median with interquartile range (IQR) or mean with standard deviation (SD) for continuous variable and as percentages for categorical variables. We compared the differences in patients characteristics at month one between the patients who followed up at 12 months post transplant with those patients who did not return for evaluation using either Pearson’s chi-square test (for categorical variables) or Wilcoxon rank sum test (for continuous variables). A linear mixed effects model with random intercepts was used to assess the change in FMD over time without and with adjustment for age, race, gender, smoking history (pack years), months on dialysis prior to transplant, cardiovascular disease, mean arterial pressure (MAP), diabetes, and estimated glomerular filtration rate (eGFR). Months on dialysis prior to transplant, eGFR, MAP, pack years, and age were included in the models as non-linear terms using restricted cubic splines. Cardiovascular disease was defined as a history of coronary artery disease, myocardial ischemia, reperfusion (coronary artery bypass or percutaneous stent), congestive heart failure, arrhythmia, stroke, or peripheral vascular disease.

A linear mixed effects model with random intercepts was used to examine the change in FGF-23 concentrations over time without and with adjustment for age, race, gender, cardiovascular disease, time on dialysis prior to transplant, and eGFR. In order to meet the normality assumption of the residuals, FGF-23 concentrations were natural logarithmic transformed. All the covariates for both models were chosen *a priori*. Analyses were performed using R, version 3.1.2 (http://www.r-project.org/). The 5 % significance level (2 sided) was used.

## Results

### Patient demographic and other characteristics

The study cohort included 149 patients who underwent evaluation at 1 month following kidney transplantation. Demographic and other patient characteristics are presented in Table 1. The cohort (*n* = 149, mean age 49, SD = 39 years) was 74 % male and 75 % White. The most common underlying etiology for ESRD was vascular disease (52 %; defined as ESRD due to diabetes- and/or hypertension-induced renal failure). Seventy-two patients (48 %) reported to be either current or former smokers, and 40 and 38 % of the cohort had a history of cardiovascular disease and diabetes, respectively. The median time on dialysis prior to kidney transplant for the cohort was 20.3 months (IQR: 6.9, 47.2). Fifteen percent of the study cohort did not require hemodialysis prior to transplant. In regards to medications that may affect the progression of endothelial function, only 3 % (4/149) of the cohort were taking angiotensin converting enzymes inhibitors and angiotensin receptor blockers, 4 % (6/149) were taking a vasodilator, 7 % (11/149) were taking an alpha blocker, and 20 % (29/149) were taking a statin. Throughout the two-year study period, there was one major cardiac event (myocardial infarction) that occurred between the 12 and 24-month follow up. There were six reported events of angina (three in the perioperative period prior to the 1 month evaluation, two events prior to the 12 month follow up, one event prior to the 24 month follow up) and one dysrhythmic event (occurred prior to the 12 month follow up).

**Table 1:** Characteristics of the kidney transplant cohort at one-month post-transplantation

Characteristic	*N* = 149
Age at transplant (years)	49 (40, 59)
Race	
White	112 (75 %)
Black	34 (23 %)
Other	3 (2 %)
Sex (male)	111 (74 %)
BMI	27.3 (23.5, 30.5)
Tobacco use	
Never	77 (52 %)
Current	14 (9 %)
Former	58 (39 %)
Pack Years	11.5 (3, 24.0)
Cardiovascular disease^a^	59 (40 %)
Diabetes	56 (38 %)
Hypertension	142 (95 %)
Hyperlipidemia	83 (56 %)
Months on Dialysis pre-transplant	20.3 (6.9, 47.2)
Primary cause of end-stage renal disease	
Vascular Disease	78 (52 %)
Glomerular Disease	31 (21 %)
Tubulointerstitial Disease	9 (6 %)
Cystic Disease	19 (13 %)
Structure Disease	1 (1 %)
Renal Neoplasia	1 (1 %)
Other	10 (7 %)
Estimated Glomerular Filtration Rate (ml/min/m^2^)	58 (48, 69)
Mean Arterial Pressure (mm Hg)	95.7 (87.3, 103.7)
Induction Immunosuppression	
Alemtuzumab	134 (90 %)
Basiliximab	14 (9 %)
Anti-Thymocyte Globulin	1 (1 %)
Maintenance Immunosuppression	
Tacrolimus	146 (98 %)
Mycophenolate	143 (96 %)
Prednisone	81 (54 %)
Anti-Hypertensive Medication	
Alpha Blockers	11 (7 %)
Beta Blockers	88 (59 %)
Calcium Channel Blockers	77 (52 %)
ACE^b^ Inhibitors	4 (3 %)
Angiotensin Receptor Blocker	4 (3 %)
Vasodilators	6 (4 %)
Statin Therapy	29 (20 %)
Slow Graft Function	23 (16 %)
Delayed Graft Function	8 (5 %)

Values expressed as median (25^th^, 75^th^ percentiles) or number (percent)

^a^Cardiovascular disease includes coronary artery disease, myocardial infarction, coronary artery bypass grafting, percutaneous coronary intervention, congestive heart failure, arrhythmia, stroke, and peripheral vascular disease

^b^Angiotensin Converting Enzyme

**Fig. 1:**
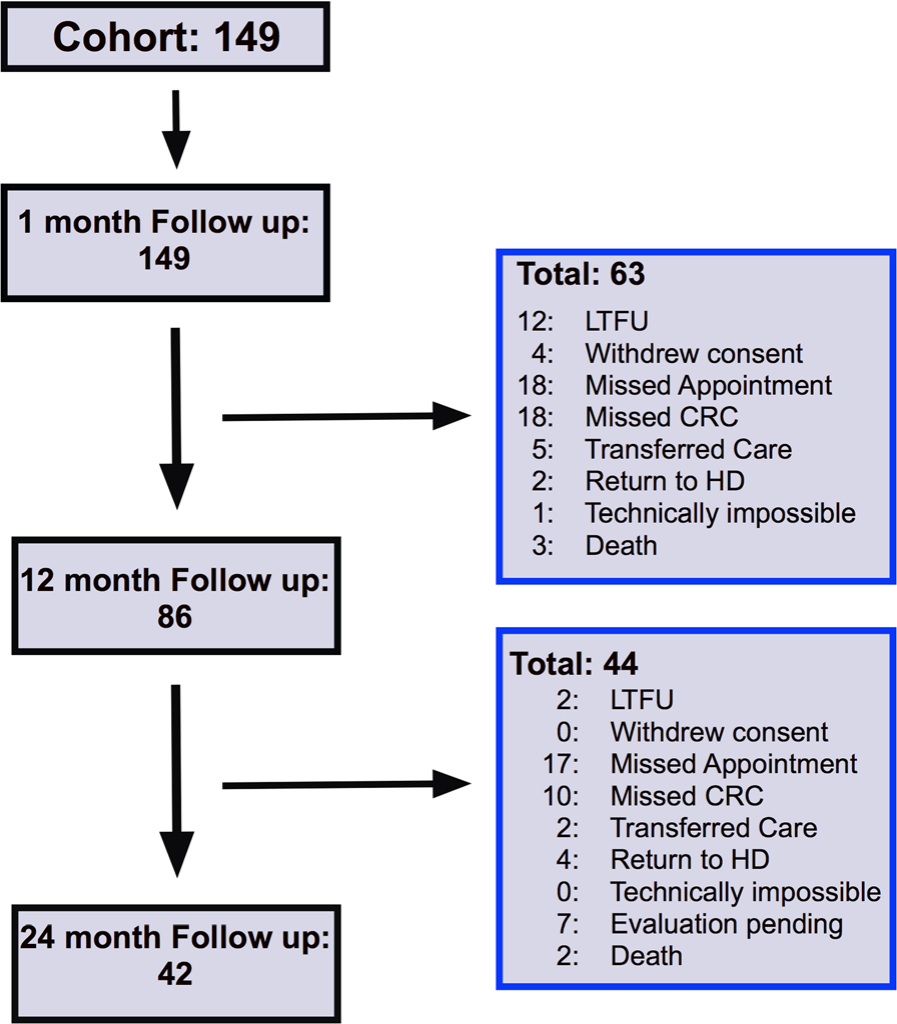
Flowchart describing the follow-up details of the study cohort of kidney transplant patients. (Abbreviations: LTFU: Lost to follow-up; CRC: Clinical research center (location of flow mediated dilation evaluation); HD: hemodialysis)

Eighty-six patients had subsequent clinical follow-up at 12 months, and 44 patients at 24 months (Fig. 1). An analysis comparing characteristics of the patients who did and did not remain in the study through the 12-month evaluation showed no statistically significant differences (For details, see Table 1 of the Supplemental Appendix). The two most common reasons (Fig. 1) for discontinuation prior to the 12-month visit were missing both the clinic appointment and ultrasound evaluation (18/63, 29 %) or missing only the ultrasound evaluation (18/63, 29 %).

### Primary outcome

The median FMD at one-month post-kidney transplant for the cohort was 6.3 % (IQR: 3.4–10.2) and at 12 months was 5.4 % (IQR: 3.1–8.5) (Table 2). Based on our definition of a normal FMD (>6.3 %), 52 % (77/149) of the population had normal endothelial function at 1 month and 49 % (42/86) at 12 months. In the unadjusted model, FMD remained stable over time (overall *P* = 0.622). Compared to month one, FMD numerically decreased by 0.56 % (95 % CI: −1.70, 0.57, *P* = 0.332) at month 12. In the adjusted model controlling for age, race, gender, smoking history (pack years), months on dialysis prior to transplant, cardiovascular disease, MAP, diabetes and eGFR, FMD remained stable over time (overall *P* = 0.532). Compared to month one in the adjusted model, FMD numerically decreased by 0.66 % at month 12, but this was not statistically significant (95 % CI: −1.81, −0.49, *P* = 0.262) (Table 3). Females had significantly increased FMD compared to males (*P* = 0.031), and FMD decreased significantly with age (*P* = 0.038).

**Table 2:** Changes in flow mediated dilation and other clinical characteristics during the study period

Parameters	
Flow Mediated Dilatation (percentage)	
Month 1	6.3 (3.4, 10.2)
Month 12	5.4 (3.1, 8.5)
Month 24	5.6 (3.5, 9.1)
Body Mass Index (kg/m^2^)	
Month 1	27.3 (23.5, 30.5)
Month 12	29.2 (26.2, 34.0)
Month 24	31.0 (26.2, 33.4)
Estimated Glomerular Filtration Rate (ml/min/1.73*m^2^)	
Month 1	58 (48, 69)
Month 12	59 (47, 71)
Month 24	57 (45, 69)
Mean Arterial Pressure (mmHg)	
Month 1	95.7 (87.3, 103.7)
Month 12	96.3 (90.8, 103.7)
Month 24	98.7 (92.5, 104.7)

Values expressed as median (25^th^, 75^th^ percentiles)

**Table 3:** Multivariable linear regression model of flow mediated dilation in kidney transplant recipients

Covariate	Effect	S.E.	95 % CI^a^	*P*
Predictors of FMD				
Age at transplant	−0.06	0.58	−1.20, 1.08	0.038
Race (reference: white)	−0.94	0.87	−2.63, 0.76	0.382
Sex (reference: males)	1.71	0.79	0.15, 3.26	0.031
Diabetes	−1.27	0.75	−2.74, 0.20	0.091
Cardiovascular Disease^b^	−0.87	0.76	−2.35, 0.62	0.252
Pack Years	−0.44	0.47	−1.36, 0.48	0.150
Glomerular filtration rate	−0.47	0.43	−1.32, 0.37	0.362
Mean arterial pressure	−0.42	0.39	−1.19, 0.34	0.976
Time on dialysis	−0.45	0.8	−2.01, 1.1	0.984
FMD 1 month -- > 12 months	−0.66	0.59	−1.81. 0.49	0.262
FMD 1 month -- > 24 months	−0.25	0.77	−1.76, 1.26	0.746
FMD 12 months -- > 24 months	0.41	0.81	−1.19, 2.01	0.614

^a^95% Confidence Interval: Lower bound, Upper bound

^b^Cardiovascular disease includes coronary artery disease, myocardial infarction, coronary artery bypass grafting, percutaneous coronary intervention, congestive heart failure, arrhythmia, stroke, and peripheral vascular disease

### Secondary outcomes

#### FMD at 24 months

**Fig. 2:**
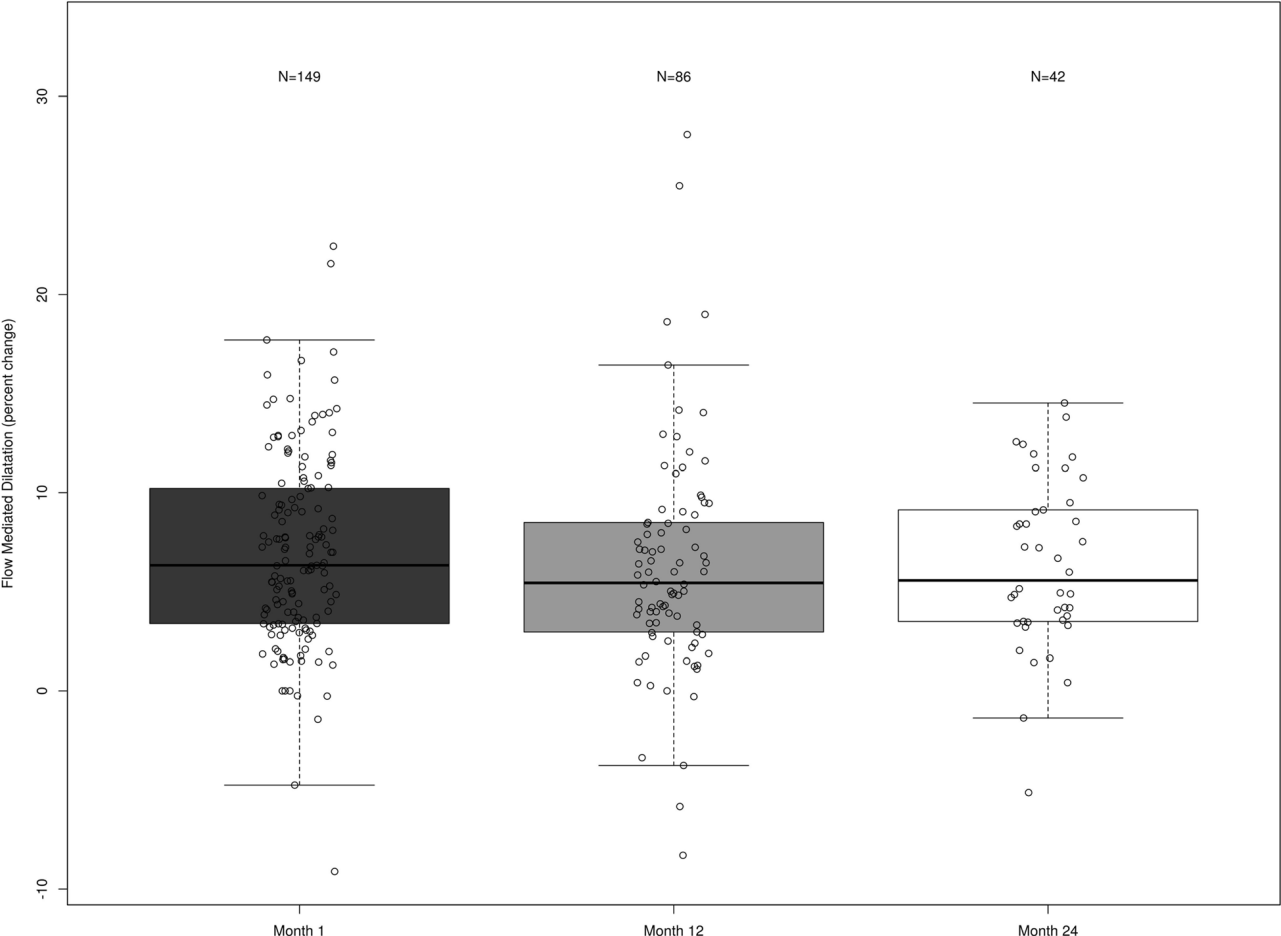
Box plot of the flow-mediated dilation measured longitudinally in the cohort population following kidney transplantation

The median FMD for the cohort at 24 months was 5.6 % (IQR: 3.5–9.1) (Fig. 2). Based on our definition of a normal FMD (>6.3 %), 50 % (22/44) of the population had normal endothelial function at 24 months. In the adjusted model, FMD remained stable from 1 month to 24 months (−0.25 %, 95 % CI: −1.76, 1.26, *P* = 0.746) and from 12 months to 24 months (0.41 %, 95 % CI: −1.19, 2.01, *P* = 0.614) post-transplantation.

#### FGF-23 levels

**Fig. 3:**
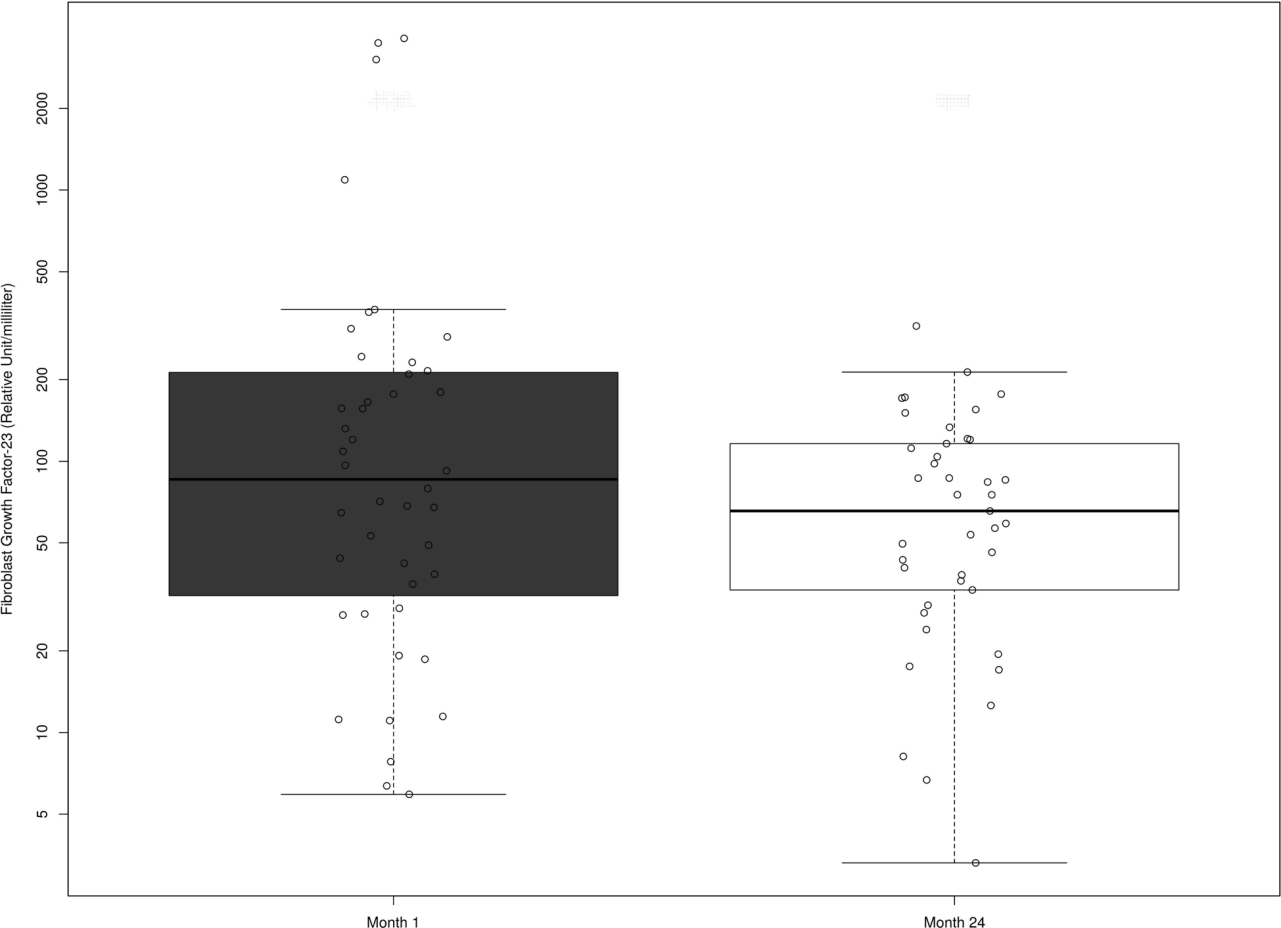
Box plot of the fibroblast growth factor 23 levels measured longitudinally in the cohort population following kidney transplantation

Forty-five patients were included in the analysis that measured FGF-23 at 1 month and 24 months follow-up after kidney transplantation. Fibroblast Growth Factor 23 decreased significantly over time (*P* = 0.02) (Fig. 3). At month 24, FGF-23 decreased 36 % (95 % CI: 7 %, 56 %, *P* = 0.024) compared to month one.

#### Flow-mediated dilation and FGF-23 level

Adjusting for age, race, gender, cardiovascular disease, time on dialysis prior to transplant and eGFR, there was no statistically significant relationship between FGF-23 and FMD following kidney transplant (*P* = 0.799). With one interquartile range (~100 picograms per milliliters) decrease in FGF-23, FMD decreased by 0.11 % (95 % CI: −0.93, 0.71, *P* = 0.799) over time from 1 month to 24 months post transplant. In addition, there was no significant association between FGF-23 and FMD at month one (*P* = 0.959) or month 24 (*P* = 0.699).

## Discussion

Endothelial dysfunction is characterized by an impaired vasodilation in response to changes in the local hemodynamic forces and vasoactive substances and can initiate the atherosclerotic pathway [29]. Since atherosclerotic disease initiated by endothelial dysfunction is a frequent cause of cardiovascular morbidity and mortality in patients with ESRD, studying changes in endothelial function by FMD may provide important information to evaluate potential interventions to address risk modification for cardiovascular disease following kidney transplantation [29]. Therefore, the aim of this study was to evaluate the long term evolution of endothelial function in kidney transplantation. We report that the improved endothelial function observed immediately post transplant is well maintained up to 2 years post transplantation. In multivariate models, only age and sex were predictors of FMD.

Endothelial dysfunction is universally present in dialysis patients, and previous studies have demonstrated a favorable change in FMD comparing pre and post-transplantation. Kocak et al demonstrated that FMD significantly improved 14 days after kidney transplant (6.69 % vs. 10.50 %, *P* < 0.001) [14]. In that study, 30 chronic hemodialysis patients with an average age of 38 years were included following living donation kidney transplant [14]. Oflaz et al evaluated endothelial function in 22 ESRD patients with an average age of 34 years and body mass index (BMI) of 23.1 kilograms/meter^2^ (kg/m^2^) [13], and reported a significant improvement in FMD from pre-transplant (6 ± 3.7 %) versus 6 months (8.3 ± 2.3 % (*P* = <0.001)) and 12 months following transplant (12 months: 12.1 ± 3.6 % (*P* = <0.001)) [13]. Yilmaz et al showed that endothelium-dependent vasodilation improved within 6 months following kidney transplant in 161 patients with an average age of 31 and average BMI of 25.3 kg/m^2^ [10]. Flow mediated dilation prior to transplant was 5.2 ± 0.8 and 6 months following transplant was 6.6 ± 0.7 (*P* = <0.001) [10].

Our study, in contrast, examines the longer-term effect of kidney transplantation on endothelial function, showing that FMD remains relatively stable from 1 month to 24 months post transplant. The patients in our cohort were initially evaluated at 1 month post transplant, a time when many of the biochemical abnormalities related to uremia have corrected and complications related to rejection or infection are rare.

Strengths of our study include the size of our cohort and duration of longitudinal follow-up. Moreover, the population in our study is older and includes a substantial proportion of patients with cardiovascular disease or diabetes, compared to the previously mentioned studies that were conducted among select groups of relatively young transplant recipients. Thus, a potential explanation for the lack of ongoing improvement in FMD in our study is the high cardiovascular risk profile of the transplant recipients. However, our study population is more reflective of the ESRD and transplant recipient population in the United States and Europe, which improves the generalizability of our findings compared to prior reports. Another strength of our study was that a single sonographer performed the FMD study in the entire cohort, which improves the reliability of the results. The advantage of using brachial artery FMD as a surrogate for coronary artery endothelial health is that the test is non-invasive with reproducible results in the same patient over time when performed by the same examiner.

A limitation of the study is the lack of a pre-transplant FMD measurement. There are reports that note FMD improves rapidly with favorable modification of cardiovascular risk factors. In the prospective, randomized RECIFE trial, FMD was shown to significantly improve in just 6 weeks (4.93+/−0.81 % to 7.0+/−0.79 % (*P* = 0.02)) in a high cardiovascular risk patient population (i.e. acute myocardial infarction or unstable angina) with hyperlipidemia who took pravastatin compared to placebo [30]. This study suggests that endothelial function rapidly improves with interventions that favorably modify cardiovascular risk, such as kidney transplantation. When compared to our study, the report by Yilmaz et al. [1] provides further insight into the potential improvement in FMD during the early post-operative period. The average FMD at 1 month after transplant in our study was 6.4 % which is similar to the FMD at 6 months following transplant (6.6 %) in the Yilmaz et al report; however, this measurement corresponded to a 27 % increase in FMD when compared to the pre-transplant FMD of 5.2 %. Without a pre-transplant FMD measurement, the immediate changes in endothelial function within the early post-operative period were not captured in our study design compared the prior reports [10, 13, 14] where the baseline FMD measurement was based on pre-transplant evaluations. However, we feel our study does provide additional information on the long-term effect of kidney transplantation on endothelial function. It is interesting to note endothelial function as measured by FMD remains stable during the first 2 years post transplantation despite the ongoing adverse metabolic effects from transplant immunosuppression and possible decline in kidney function.

Fibroblast Growth Factor-23 has been shown to be significantly elevated in patients with chronic kidney disease as FGF-23 metabolism and clearance are modified [31, 32], which subsequently improves following kidney transplantation [15]. Several studies have shown a link between elevated FGF-23 levels and increased cardiovascular risk. In a prospective study of 984 kidney transplant patients, Wolf et al showed that elevated FGF-23 levels post-operatively were independently associated with increased mortality and allograft loss [15]. Elevated FGF-23 levels have been correlated with impaired endothelial function in patients [19, 22–24]. Mirza et al demonstrated an association between higher FGF-23 levels and impaired vasoreactivity, as well as, a correlation between arterial stiffness with patients with renal dysfunction and higher FGF-23 levels [24]. Yilmaz et al reported an independent correlation of high FGF-23 and impaired FMD in patients with stage III and IV renal failure (20). The relationship between decreased FGF-23 concentrations and endothelial function following kidney transplantation is not well studied, but one report showed in 161 patients that endothelium-dependent vasodilation improved in the 6 months following kidney transplant, which corresponded to a significant decrease in FGF-23 levels [10]. In our study, there was no significant association between FGF-23 levels and FMD over 24 months following kidney transplant despite a significant reduction in FGF-23 concentration over the two-year period. The difference in outcomes between the two studies could be a result of the shorter follow up time in the Yilmaz et al report (6 months versus 24 months, respectively) and, as previously stated, the dissimilarity in the demographic profiles.

Further limitations of our study include the high percentage of patients who did not complete the follow up. A sensitivity analysis did not show any significant demographic or clinical differences between patients who did not return for the 12 month follow up compared to patients who completed the study, suggesting that any bias is likely to be minimal, though in characteristics not examined cannot be ruled out. Another limitation of our study is that measurements for maximal FMD were obtained at set time points of 60 and 90 s. Without a continuous evaluation, the maximum diameter change secondary to reactive hyperemia may not have been captured with these set time point measurements. In addition, FMD can be affected by temperature, foods, drugs, sympathetic stimuli, environmental factors, and patient’s positions, which can vary over time at each evaluation. The study protocol attempted to minimize this variation by asking all patients to not consume tobacco, food or liquids for 8 h prior to the evaluation. In addition, cardiovascular medication such as angiotensin converting enzyme inhibitors and statins that could affect endothelial function were not controlled for in the study cohort.

## Conclusions

In a single center, prospective study of medically stable, kidney transplant recipients who were initially evaluated at 1 month following surgery, FMD remained stable over a 24 month follow up period. Consistent with previous work, FGF-23 levels significantly decreased following kidney transplant, however this decrease was not associated with improvement in FMD. The stable FMD in our study cohort suggests that the improvement in endothelial function is maintained up to 2 years post transplantation. However, the lack of continued improvement in endothelial function may contribute to the persistently elevated cardiovascular risk in transplant patients compared to the general population despite improved renal function. Further prospective research regarding endothelial function following kidney transplant would provide important insight into possible mechanisms whereby transplantation impacts cardiovascular health, which would allow interventions aimed at the prevention and reversibility of cardiovascular diseases.

## Abbreviations

BMI: Body mass index
eGFR: Estimated glomerular filtration rate
ESRD: End stage renal disease
FGF-23: Fibroblast growth factor
FMD: Flow mediated dilation
IQR: Interquartile range
kg/m^2^: Kilograms/meter^2^
MAP: Mean arterial pressure
SD: Standard deviation
VUMC: Vanderbilt university medical center
